# Potential Diagnostic Role of Hepcidin in Anemic Patients Affected by Inflammatory Bowel Disease: A Systematic Review

**DOI:** 10.3390/diagnostics14040375

**Published:** 2024-02-09

**Authors:** Fabiana Ferrari, Mattia Carini, Isabella Zanella, Giorgio Treglia, Gaetano Luglio, Roberto Bresciani, Giorgio Biasiotto

**Affiliations:** 1 Pediatrics, Mother’s and Baby’s Health Department, Poliambulanza Foundation Hospital Insitute, 25124 Brescia, Italy; 2 Department of Molecular and Translational Medicine, University of Brescia, 25123 Brescia, Italy; 3 Highly Specialized Laboratory, ASST Spedali Civili di Brescia, 25123 Brescia, Italy; 4 Section of Genetics and Cytogenetics, ASST Spedali Civili di Brescia, 25123 Brescia, Italy; 5 Clinic of Nuclear Medicine, Imaging Institute of Southern Switzerland, Ente Ospedaliero Cantonale, 6501 Bellinzona, Switzerland; 6 Faculty of Biology and Medicine, University of Lausanne, 1011 Lausanne, Switzerland; 7 Faculty of Biomedical Sciences, Università della Svizzera Italiana, 6900 Lugano, Switzerland; 8 Endoscopic Surgery Unit, Department of Medical and Surgical Gastrointestinal Disease, “Federico II” University, 80131 Naples, Italy

**Keywords:** hepcidin, ferritin, biomarkers, inflammatory bowel diseases, anemia, systematic review

## Abstract

Background: Anemia is the main extraintestinal comorbidity of Inflammatory Bowel Disease (IBD). Differentiating the type of anemia in these disorders is still a challenge. Hepcidin could be a promising biomarker to identify iron deficiency anemia (IDA), anemia of chronic disease (ACD) and the concomitant presence of both IDA and ACD. Methods: To evaluate the potential role of hepcidin dosage in the management of anemia in IBD patients, we performed a systematic review by a comprehensive literature analysis of original papers reporting the dosage of hepcidin in IBD patients. In all the articles reviewed, the dosage of ferritin was reported, and the correlation between hepcidin and ferritin has been used to compare these two biomarkers. Results: A total of 12 articles concerning the dosage of hepcidin in IBD were included, comprising in total of 976 patients. The results of the hepcidin values in IBD patients when compared with controls were conflicting. In fact, four articles described an increase in this biomarker, three showed a decrease and five did not find significant differences. The correlation with ferritin was positive and significant. In three studies, some differences between hepcidin dosages and ferritin levels indicate a possible role when IDA and ACD could be present at the same time. Conclusions: Considering the contradictory data of the studies, the diagnostic role of hepcidin as a biomarker remains elusive in IBD patients. These differences could be due to the clinical characteristics of the patients enrolled that should be better defined in the future. A suitable clinical trial should be designed to outline the possible role of hepcidin in differentiating IDA, ACD and concomitant IDA and ACD in IBD patients. At the moment, ferritin still remains the best marker to diagnose these conditions, in addition to hemoglobin, transferrin saturation and CRP as recommended by the ECCO guidelines.

## 1. Introduction

Inflammatory Bowel Disease (IBD) is a group of chronic inflammatory diseases of the gastrointestinal apparatus that can be divided into Crohn’s Disease (CD) and Ulcerative Colitis (UC). These chronic disorders can evolve in unpredictable ways, adding complications to the clinical management of the patients. The main histopathological characteristic of CD is a discontinuous, transmural inflammatory state that can manifest in every part of the gastrointestinal tract, while UC is characterized by a more superficial and continuous inflammation of the colon wall that involves predominantly the mucosal and submucosal layers [1]. The etiopathogenesis is multifactorial but is yet to be clarified; the leading causes seem to be related to the genetic and immunological background, but also various environmental factors should be considered [2,3]. Recent data reported that more than two million inhabitants of North America and about the same number of Europeans are affected by IBD, and these numbers are expected to grow in the next few years [4].

Anemia is a frequent comorbidity in IBD: it adds a heavy burden to this condition and has been associated with a decreased quality of life and impaired cognitive functions. Anemia in IBD could be due to a single pathological condition or could be the result of different causes classified by the European Crohn’s and Colitis Organization (ECCO) in iron deficiency anemia (IDA), anemia of chronic disease (ACD) and anemia associated with the lack of nutritional cofactors such as B-12 vitamin or folic acid [5,6]. Additional causes may be pharmacological treatments such as methotrexate, thiopurines and 5-aminosalicylic acid [7]. The prevalence of anemia in IBD remains difficult to determine and can range between 36% and 90% [3,8]. The risk of developing anemia may vary depending on age, particularly among patients between 18 and 25 years and those who are over 65 years. The type of anemia seems to correlate with the type of IBD, as IDA appears to be more present in UC patients, while ACD is more frequent in CD [9]. However, anemia in IBD could also be caused by iron deficiency or inflammation. The blood parameters in IDA are characterized by low serum iron, low serum ferritin, low transferrin saturation and increased total iron binding capacity (TIBC). ACD is often associated with inflammatory diseases such as autoimmune diseases, heart diseases and kidney diseases. ACD is generally normochromic, slightly microcytic or normocytic; iron levels could be normal, and ferritin values could be normal or increased [10]. The process of differentiation between IDA and ACD is still a clinical challenge in IBD, where these two forms of anemia could coexist.

Hepcidin is a peptide mainly produced by the liver and is genetically highly conserved in mammals; it contains eight cysteines forming four disulfide bonds in a single hairpin structure. Hepcidin is believed to function like a hormone that regulates iron metabolism responding to iron levels in the body and modulating iron absorption and compartmentalization. When iron levels and storage are high, hepcidin is synthesized mainly by the liver, and it blocks the iron absorption and the release from the stores, decreasing sideremia. The molecular mechanism consists in the binding of hepcidin to ferroportin, the only known iron exporter from cells, thus inducing its internalization and degradation. As a result, ferroportin is destroyed and the total amount of iron in the body is restricted because iron contained in enterocytes, macrophages and hepatocytes cannot be released in the blood and bind to transferrin. On the contrary, when the body needs iron such as in iron deficiency, anemia, hypoxia or when it is necessary to increase erythropoietic activity, the synthesis of hepcidin is inhibited, allowing iron absorption from the enterocytes and iron release from the spleen macrophages and from the stores [11,12]. Mutations in genes codifying for proteins involved in the process of iron sensing can cause an iron overload pathology known as Hereditary Hemochromatosis [13,14,15]. Iron metabolism is also conditioned by inflammation, and Interleukin 6 (IL-6) has been described to be an important inducer of hepcidin synthesis via the JAK/STAT3 inflammatory pathway [16,17]. This mechanism is part of innate immunity, and it decreases both the amount of iron in the blood and in the extracellular fluid, thus subtracting it from potential pathogens but causing anemia of inflammation. In this inflammatory context, hepcidin is regulated as a type II acute-phase protein [16].

Recently, different authors have studied hepcidin levels in the blood of IBD patients to verify its potential as a noninvasive biomarker able to connect inflammation and iron metabolism, especially in the presence of anemia [18,19,20,21,22,23,24,25,26,27,28,29].

The aim of this systematic review is to focus on the possible role of hepcidin in differentiating IBD patients affected by anemia and the comparison of its levels with those obtained by testing serum ferritin.

## 2. Material and Methods

### 2.1. Protocol

This systematic review was prepared by referring to the “Preferred Reporting Items for a Systematic Review and Meta-Analysis” (PRISMA 2020 Statement) [30]. The complete PRISMA checklist is contained in the Supplementary Materials (Table S1).

The main questions of the review are the following: (a) Can hepcidin quantification help discriminate the type of anemia in patients affected by Inflammatory Bowel Disease? (b) Can hepcidin add more information than ferritin?

The literature search strategy has been created using the Population, Intervention, Comparator, Outcomes (PICO) framework. The parameters of the study inclusion were the dosage of serum hepcidin in anemic patients affected by IBD in comparison to the values of serum ferritin (comparator). The accuracy of the hepcidin values in patients affected by iron deficiency anemia (IDA) and in anemia of chronic disease (ACD) were used as outcomes.

Three Reviewers (G.B., F.F. and M.C.) carried out the literature research, the quality evaluation of the studies and the data extraction.

### 2.2. Literature Search Strategy and Information Sources

The authors comprehensively checked the literature using the following bibliographic databases: PubMed/MEDLINE and Cochrane Library. The research included papers that explored hepcidin, ferritin and other iron metabolism biomarkers in patients affected by IBD.

The algorithm to extract the articles was a combination of the following terms: hepcidin AND (“IBD OR Inflammatory bowel disease*”); Hepcidin AND Ferritin AND “Inflammatory Bowel Disease*”. No language restrictions were applied in the research. The References of the articles were considered to include additional papers of interest. The evaluation of ongoing studies was performed by consulting the ClincalTrials.gov database (https://clinicaltrial.gov; last update was made on 31 December 2023).

### 2.3. Eligibility Criteria

All the clinical studies/Original Articles containing hepcidin and ferritin dosage in IBD patients affected by anemia were taken into consideration for their inclusion in the Systematic Review. In addition, the presence of a control cohort was evaluated before the inclusion. Only human studies were considered for the data collection. Only studies containing a correlation between hepcidin and the comparator ferritin were included. Case Reports, Letters, Editorials, Comments and Preclinical Studies were excluded from the analysis.

### 2.4. Selection Process

Three authors (G.B., F.F. and M.C.) made an independent evaluation of the titles and the abstracts of the articles. The eligible papers were selected based on the predetermined inclusion and exclusion criteria. The reasons for the choice were specified.

### 2.5. Data Collection Process and Data Extraction

The authors mentioned the above-extracted data from the selected studies by analyzing full texts, figures and tables to obtain general information about each study (Authors, Country, publication year, study plan and funding); patient information (number, age, gender, diagnosis and laboratory tests); tests of biomarkers (hepcidin tests, ferritin tests, Hemoglobin quantification, other iron parameters and inflammatory biomarkers tests).

### 2.6. Quality Assessment (Risk of Bias Assessment)

The risk of bias was evaluated by using QUADAS-2 for a single study in order to verify the applicability of the Review argument. This method permitted us to assess the quality and the accuracy of the biomarker tests in the selected studies [31]. Three Authors evaluated the grades of the studies in the systematic review analyzing four domains (patient selection, index test, reference standard, and flow and timing) to estimate the risk of bias in three areas about the applicability (patient selection, index tests and reference standards).

## 3. Results

### 3.1. Literature Search and Study Selection

**Figure 1:**
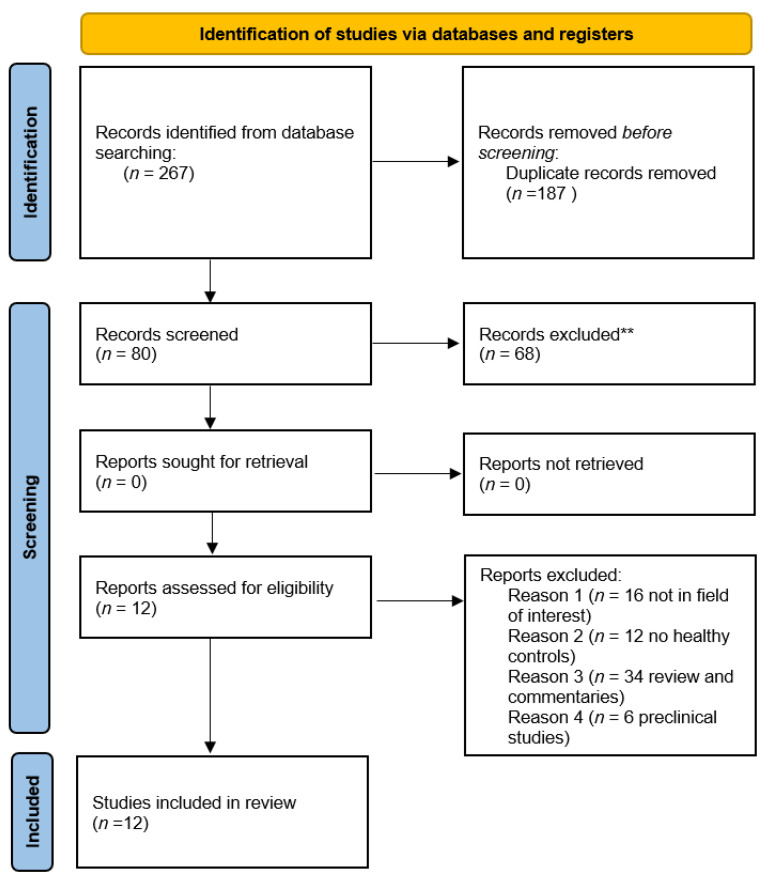
A summary of the section process of the studies included in the systematic review (Page et al., 2021) [30]. ** The reasons of the exclusion of the records are reported below in dedicated box.

The research in the scientific literature was concluded on 31 December 2023 and included 80 articles. After the revision of these works based on the predetermined parameters described in the Material and Methods section (inclusion and exclusion criteria), 68 articles were evaluated as unsuitable for this systematic review, including 34 reviews, editorials and comments to articles or case reports; 16 were not in the area of interest, 12 did not provide a useful cohort of controls and 6 were pre-clinical studies. Only 12 were considered eligible for the systematic review at the conclusion of the revision process [18,19,20,21,22,23,24,25,26,27,28,29]. The analysis of the references of the selected articles did not add any additional suitable articles to the final selection. The selection process is summarized in Figure 1.

### 3.2. Study Characteristics

The characteristics of the studies included in the final evaluation were reported in Table 1, Table 2 and Table 3. The comprehensive analysis included 976 IBD patients and 402 Controls (the study by Stojkovic Lalosevic et al., 2020 [24] mentions the healthy controls but does not specify their number) The articles were published between 2009 and 2023 in Europe, the USA, China and Israel. A total of 10 studies were conducted with a prospective/monocentric design [18,20,21,22,23,24,25,26,28,29], one study was cross-sectional and monocentric [19] and one study was observational and multicentric [27] (Table 1).

The characteristics of the patients are described in Table 2. The number of patients in the studies was between 10 and 247. The mean or median ages of patients ranged from 12.6 to 49 years with a percentage of females between 42% and 70%. The female percentage was not shown in the study by Arnold et al. [22]. The control cohorts of the studies had a number between 8 and 102, while the mean/median age and female percentages ranged, respectively, from 10.88 to 49 and from 19.61% to 62.5%. Some of these data were lacking in these studies [18,24,27]. All the articles analyzed tested hepcidin concentrations both in patient and control blood samples. The IBD cohort was subdivided into CD and UC patients in all the studies except for the study by Ben-David et al. [25]. Furthermore, almost all the studies classified the patients as anemic and non-anemic apart from Moran-Lev et al. [20]. Hepcidin was quantified using different laboratory methods; ELISA marketed by different companies [18,20,21,23,24,29], Mass spectrometry [19,26] and RIA [22]. Three studies did not specify the analytical method used to evaluate hepcidin concentration [25,27,28] (Table 2). Generally, hepcidin quantification was expressed in ng/mL. When the concentration was expressed in nM, the values were converted to ng/mL to facilitate the comparison of this systematic review [19,26].

In all the studies, the diagnostic performance of blood hepcidin was compared with the ferritin concentration. In addition, the hepcidin levels were compared with inflammation markers such as CRP in ten studies [18,19,20,21,24,25,26,27,28,29], ESR in three articles [21,24,29] and IL-6 in two papers [20,22]. Iron metabolism was also explored using other biomarkers in addition to hepcidin and ferritin, such as sTFR, which was tested in six studies [19,22,25,26,28,29].

Considering the asymmetrical distribution of hepcidin values in the cohorts of patients and controls, the descriptors of the statistical variables were median and range intervals in most of the studies. Because of this fact, a meta-analysis could not be performed.

### 3.3. Risk of Bias and Applicability

**Figure 2:**
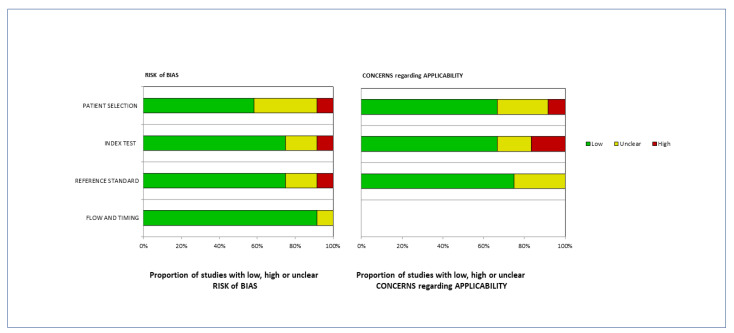
An assessment of quality was evaluated using the QUASAD-2 instrument. The studies were classified based on the risk of bias (Low, High and Unclear) or applicability concerns for different issues reported in the ordinate axis. The percentage of the studies was reported in the abscissa axis. The results of this evaluation showed that less than 20% of the selected studies were spoiled by a high risk of bias.

The Authors analyzed the data reported in the studies and carried out the risk of bias and concerns considering the applicability of the selected articles using the QUASAD-2 tool. The results of this evaluation are reported in Figure 2.

### 3.4. Results of Individual Studies (Qualitative Synthesis)

The dosage of hepcidin could be a significant marker to investigate anemia in IBD. All the selected articles investigated hepcidin in patients and in controls and compared this biomarker to others referred to iron metabolism and inflammation. Serum ferritin shows the best characteristics to link the world of iron metabolism and inflammation, because it can be used to evaluate iron storage, but it is also known as an acute-phase protein. These features are shared with hepcidin, which increases both when iron storages are replete and under various inflammatory stimuli. This is the reason why ferritin can be used as a good comparator for the diagnostic performance of hepcidin. In addition, ferritin is recommended by ECCO guidelines as the biomarker to evaluate iron metabolism in anemic IBD patients.

The outcome of the analysis of the selected studies is that the utility of hepcidin is unclear. In some articles, its values were increased more in patients than in controls [18,19,20,21]. In some others, the opposite occurred [22,23,24], or no difference was observed [25,26,27,28,29] (Table 3).

Arnold et al. reported that hepcidin levels were significantly lower in the serum of IBD patients when compared with healthy controls. When the patients were stratified for the presence or absence of iron deficiency anemia and referred to controls, the hepcidin levels remained still low (respectively, mean ± SEM 6.81 ± 1.2 and 4.14 ± 0.72 vs. 15.3 ± 3.14). No differences were mentioned between UC and CD patients. The hepcidin levels positively correlated with ferritin and IL-6 values [22].

Serum hepcidin and prohepcidin in IBD patients were studied by Oustamanolakis et al. Unlike the previous study, hepcidin levels in IBD patients were higher than in healthy controls. Both UC and CD showed the same trend when compared with controls (respectively, in median and range, 73.3, (16.5–736) and 70.9, (16.7–306.9) vs. 47.0, (8.6–340.2)), but only in UC patients’ hepcidin correlated with disease activity. The Authors found a strong correlation with the main comparator ferritin, whose values were probably inverted in the table of the article [18].

The interesting study by Bergamaschi et al. dosed two isoforms of hepcidin (hepcidin-25 and hepcidin-20) using mass spectrometry. In this study, the quantification of hepcidin was in nM, and a conversion to ng/mL was made to make the values comparable with those reported in the other works. The results of this study did not show any difference between the IBD patients and healthy controls (respectively, hepc25 values in median and range, 5.24, 3.12–8.84 vs. 10.09, 7.98–10.09). In the analysis of the subgroups, the Authors found that patients with anemia of inflammation (AI) had higher levels of hepcidin than patients with IDA or patients with a contemporary presence of iron deficiency anemia and inflammation. In this work, a cut-off value of 5.85 ng/mL (2.1 nM) was proposed to differentiate the 85% of patients affected by IDA (even in the presence of inflammation) from patients with AI. A strong correlation with ferritin values was shown [26].

Mecklenburg et al. enrolled the largest cohort of IBD patients (130 CD and 117 UC for a total of 247 patients) in their retrospective study. The patients were separated into five subgroups based on the following criteria: active or inactive disease, anemia and iron deficiency. In this work, hepcidin levels correlated only with ferritin but not with the other inflammation markers. Furthermore, hepcidin strongly correlated with ferritin, and all patients with low ferritin had low levels of hepcidin. Patients and controls with similar levels of ferritin had similar hepcidin values. The biochemical parameters of the cumulative cohorts of patients and controls were not reported in the article [27].

The blood level of hepcidin, the disease activity and the Iron Load Test (ILT) were studied in a cohort of IBD pediatric patients and suitable controls by Martinelli et al. The interesting observation of this study is that it showed a higher value of hepcidin in patients with active disease (Patients and Controls, respectively, mean ± SD (range) 12.55 ± 24.0 (1.5–108.5) ng/mL vs. 5.86 ± 7.25 (1.5–31.5) ng/mL). The increase in hepcidin levels was significant when compared to both controls and patients with coeliac disease. The ILT test was pathological when active disease was present. The comparison between hepcidin and ferritin evidenced a strong correlation, and it is noteworthy that the hepcidin values increased with a normal ferritin level in 53.8% of the patients affected by combined IDA and ACD [19].

Krawiec et al. studied hepcidin values in a pediatric cohort of IBD patients and in pediatric controls comparable for age. The hepcidin levels in blood were significantly decreased when compared with controls (respectively, mean ± SD, median (range) 5.9 ± 5.08, 4.76 (0.27–32.93) vs. 10.00 ± 10.04, 7.15 (0.004–44.78)). The hepcidin results were comparable in CD and UC. Anemic IBD patients showed significantly decreased levels of hepcidin when compared to healthy controls. Hepcidin levels were non-significant in anemic and non-anemic IBD patients, as well as in non-anemic patients and healthy controls. Among the various parameters of iron metabolism and inflammation, only ferritin was strongly associated with the hepcidin values [23].

Another case study by Moran-Lev et al. examined children affected by IBD. After the characterization of iron parameters and inflammatory markers, the Authors studied the effect of vitamin D in ameliorating the clinical picture. Serum hepcidin was significantly higher when compared with the controls at the time of enrolment (respectively, median (Interquartile range) 34.2 (21.3–44.7) vs. 13.3 (3.5–23.4)). Interestingly, no difference was found in the ferritin levels of IBD patients and controls [20].

A study by Shu et al. added some information by analyzing patients with inactive disease after treatment with a biological anti-TNF-alpha drug. In this study, hepcidin levels were higher in IBD patients with active pathology than in those with remitting IBD and healthy controls, but the numerical values of the dosages were not reported in detail. In addition, the hepcidin values positively correlated with the severity of anemia and were higher in anemic IBD patients, while serum ferritin did not significantly change between anemic and non-anemic patients. Hepcidin levels positively correlated with serum ferritin in IBD patients, both affected by UC or CD. [21].

Aksan et al. investigated both iron and inflammatory parameters to assess the oral iron absorption capacity in IBD patients and healthy controls. Although the method used to quantify hepcidin was not specified, in this study, there seemed to be no difference in hepcidin levels in patients with either active or non-active disease and healthy controls (respectively, median (range) 21.95 (11.35–158.5) vs. 26.45 (16.80–56.2). This study also confirmed a good correlation between hepcidin and ferritin levels [28].

Also, Stojkovic Lalosevic et al. tried to verify if hepcidin could be a useful marker of anemia and disease activity in IBD patients. The Authors found that hepcidin levels were lower in IBD patients than in controls (respectively, mean ± SD 6.4 ± 2.42 vs. 9.77 ± 2.71). The serum hepcidin values were similar in CD and UC patients and significantly lower in patients with IDA when compared with patients affected by ACD. Serum ferritin showed the same performance as hepcidin, with higher levels in controls than in IBD patients. The correlation between hepcidin and ferritin was significant and positive [24].

Ben-David et al. compared a small cohort of children affected by IBD with three other groups of patients affected by different types of anemia. Despite the lack of a real healthy control group, the comparison was still interesting. The IBD patients carried hepcidin levels significantly lower than the levels of patients with anemia of inflammation but comparable with the levels of patients affected by anemia caused by celiac disease who were used as controls (IBD patients vs. celiac disease patients, respectively, mean ± SD 6.8 ± 3.6 vs. 6.6 ± 5.9). Moreover, when comparing IBD patients with IDA patients, the hepcidin levels were lower in the IDA cohort without reaching statistical significance. These data suggested a combined effect of iron deficiency and inflammation. The correlation with ferritin levels was positive and significant [25].

An interesting study was published also by Loveikyte et al. They analyzed hepcidin and iron metabolism in IBD patients in therapy with Vedolizumab and Infliximab. In particular, the baseline data of the patients were very useful for this systematic review. The hepcidin values were lower in patients compared to controls without reaching statistical significance (respectively, median (range) 13.52 (4.85–28.72) vs. 21.19 (9.84–33.29). Iron deficiency was present in almost all the patients who had hepcidin values below the median value. Therefore, hepcidin could accurately differentiate this condition. The Authors concluded that iron deficiency was the main factor that influenced hepcidin levels, even when inflammation was present. The analysis of oxidative stress indicated that the parameters related to this condition were increased when hepcidin was low. The hepcidin values correlated positively with ferritin levels [29].

Summarizing the findings concerning hepcidin levels in the IBD patients reported in the studies analyzed, we could observe that hepcidin levels were higher than in controls in four studies [18,19,20,21], comparable in five [25,26,27,28,29] and lower in three others [22,23,24].

## 4. Discussion

Anemia is one of the comorbidities of IBD that have a significant impact on the clinical status of patients. This condition adds a heavy burden to these patients, causing general weakness, mood disorders, impaired cognitive functions and poor quality of daily life. Anemia in IBD is caused by different conditions often present at the same time, such as inflammation of gastrointestinal mucosa, defective absorption of vitamin B12 and/or folate and chronic intestinal bleeding. Another important condition responsible for at least half of the cases of anemia is iron deficiency, which may justify iron supplementation in the treatment of these patients. Furthermore, anemia is correlated with a worse prognosis, various complications and more severe forms of IBD [32]. The treatment of anemic patients should be recommended in IDA. The recovery of normal values of hemoglobin and iron stores requires a long treatment. The satisfactory therapeutic response is typically represented by an increase in hemoglobin equivalent to 2 g/dL in a period of 4 weeks and the achievement of more than 30% of transferrin saturation with serum ferritin values greater than 10 ng/mL [2,33]. The ECCO guidelines recommended oral iron treatment for IDA patients with normal CRP and hemoglobin values greater than 10 g/dL. Oral iron is not entirely absorbed, and the remaining part is held responsible for causing alterations in the microbiota and mucosal damage. Additionally, an increase in pro-inflammatory cytokines was shown in IBD animal models. The administration of an intravenous iron formulation is recommended in patients with severe anemia with hemoglobin values below 10 g/dL [2,6,33]. Some nutraceutical molecules contained in functional foods are under evaluation for their properties to ameliorate bowel inflammation and iron absorption [34,35].

The amount of iron stored in human adults is usually around 3–4 g. The iron homeostasis in the body is maintained by balancing iron absorption and the physiological iron loss mainly by desquamation of the epithelia. Multiple factors may cause iron deficiency in IBD patients, such as bowel bleeding related to inflammation, impaired absorption caused by the Short Bowel Syndrome, reduced appetite and impaired internal iron recycling accomplished by macrophages [3].

Hepcidin was discovered in 2001 as a peptide synthesized by the liver that shows a fundamental role in regulating systemic iron metabolism. The effect of hepcidin expression is to decrease iron absorption, to inhibit the release of iron from the liver stores and from the catabolism of red blood cells by macrophages. Consequently, replete iron stores and inflammation increase hepcidin production, while iron shortage and increased erythropoiesis inhibit its expression. Additionally, IL-6 and other inflammatory cytokines induce the expression of hepcidin by the pathway JAK2/STAT3, causing anemia of inflammation [36,37].

Practically all the biomarkers used in routine laboratory testing to evaluate iron parameters are influenced by inflammation, and this is an obstacle in the evaluation of the patient’s iron stores and metabolism.

Serum ferritin showed a higher accuracy in diagnosing IDA and can be used to distinguish IDA from ACD. In fact, blood ferritin is usually decreased in IDA and increased or normal in ACD. The ECCO guidelines established that the ferritin value in IBD patients without clinical exacerbation should be below 30 ng/mL [33]. Ferritin values in the blood of anemic patients up to 100 ng/mL may be a sign of iron deficiency caused by an inflammatory state [38]. However, the role of ferritin in the diagnostic process of IDA with concomitant ACD was not completely satisfactory, and a threshold value with enough sensitivity and specificity could not be established [39,40]. In almost all the studies analyzed, hepcidin and ferritin showed a strong correlation, evidencing a coordinated control of their expression in IBD patients also during inflammation. In this last case, common triggers could probably be due to the same proinflammatory cytokines such as IL-6. However, some particularities in their movements in IBD patients might be noteworthy (Table 3).

Despite the relevant number of patients obtained by putting together the enrolments of all the studies, it is difficult to make a synthesis summarizing the observations about the use of hepcidin as a biomarker related to iron metabolism and anemia in IBD patients. In brief, it was noticed that the levels of hepcidin were contradictory and difficult to understand. In fact, serum hepcidin levels were higher than in controls in four studies [18,19,20,21], comparable in five works [25,26,27,28,29] and lower in three articles, Arnold [22,23,24]. Three studies regarded pediatric patients, but also, in these studies, the results remained contradictory with two cases evidencing an increase in serum hepcidin [19,20], while one showed a decrease [23]. Unfortunately, the asymmetric statistical distribution of the values of hepcidin in the blood made it impossible to perform a meta-analysis also when the distribution was treated as symmetric.

However, we could hypothesize that these contradictory results could depend on the heterogeneity of the patients enrolled in the different studies. This assumption may be confirmed by considering the correlation between hepcidin and ferritin, which was generally good and consistent regardless of the hepcidin movement in the blood and the type of the patients chosen.

It should be also considered that some difficulties in comparing the various studies could be due to the different methods applied to detect hepcidin in the blood. Enzyme-linked immunoassays (ELISA) were used in seven studies [18,20,21,23,24,27,29], mass spectrometry was used in two [19,26] and radioimmunoassay (RIA) was used in one [22]. In Aksan et al. and Ben-David et al. [25,28], the method was not specified. Although mass spectrometry is widely known as the gold standard method, immunoassays have shown very good performance in clinical testing. The main diagnostic difference between these two methods was the possibility of mass spectrometry to specifically measure the hepcidin-25 isoform, while immunoassays generally do not discriminate among isoforms. The correlation among the various methods remains satisfactory with some differences In measured hepcidin values that tend to be higher in immunoassays [41,42,43]. Because of the lack of a universally acknowledged method to dose hepcidin in blood, well-defined reference values are still to be obtained, and their absence could make the interpretation of data quite challenging. However, the control cohorts may help to evaluate the movement of blood hepcidin in the IBD patient’s cohort.

Even though hepcidin’s role in the differentiation of anemia in IBD is not fully clarified, it is known that inflammation in the absence of iron shortage increases hepcidin levels [44]. In the presence of iron deficiency, instead, the hepcidin value remains low even in the course of an inflammatory state, emphasizing the importance of safeguarding iron absorption. The concomitant presence of opposite stimuli such as inflammation and iron deficiency could interfere with the regulation of hepcidin expression in IBD patients, altering the expected fluctuation of the hepcidin level In the blood. Animal models showed that when iron shortage and inflammation are present at the same time, the deficit of iron seems to control the downregulation of hepcidin expression, eliminating the stimulatory effect of inflammation [45,46]. Furthermore, in patients with stable heart failure and anemia, hepcidin expression was regulated mainly by the amount of iron in the body rather than by inflammation [47]. Bergamaschi et al. confirmed this observation in IBD patients with concomitant IDA and ACD. In their study, the Authors proposed a cut-off hepcidin value of 2.10 nM (5.85 ng/mL), with ferritin values up to 190–200 ng/mL, to discriminate patients affected by iron deficiency with or without inflammation (87% sensitivity and 87% specificity) [26]. Martinelli et al. confirmed a good correlation between hepcidin and ferritin, which was in agreement with the observation that ferritin was the main biomarker correlated with hepcidin levels. These Authors found that 53.8% of the patients with concomitant IDA and ACD showed normal values of ferritin and increased hepcidin levels when compared with IDA patients, and they concluded that ferritin alone was not sufficient to discriminate the type of anemia in IBD patients [19]. Serum hepcidin significantly increased in the pediatric IBD patients when compared to the controls, while no differences were found in the ferritin levels between these two cohorts in the study by Moran-Lev et al. These data could be in line with those reported by Martinelli et al. considering the increased value of CRP and IL-6 and the decreased hemoglobin value, but some additional data about disease activity would help to better compare the results of these two studies [20].

Further studies based on the evaluation of the iron status in the bone marrow aspirates may be necessary to correlate hepcidin levels in blood and body iron stores. The optimal result could be to determine a hepcidin cut-off value able to distinguish IDA and ACD and that differentiates functional iron deficiency in IBD patients. Hepcidin could also be useful in predicting the response to oral iron administration or parenteral treatment, thus adding further value to its laboratory determination. These options could make hepcidin an important biomarker in addition to ferritin in the complex world of iron metabolism in IBD. Further studies with an adequate number of patients are needed to better define its potential role, especially when IDA and ACD anemia are present in IBD patients. In the meantime, ferritin remains the main reference marker as indicated by ECCO guidelines with hemoglobin (CBC), transferrin saturation and CRP [33].

This systematic review shows some limitations. The characterization of the IBD patients in the studies was not sufficiently detailed, and this fact complicated the interpretation of the causes of the hepcidin movements. The heterogeneity of the cohorts of patients was evident also when age was considered; in fact, three studies regarded pediatric populations [19,23,25]. Moreover, the methods used to dose hepcidin were different in the studies analyzed and could cause some heterogeneity in the results. Lastly, since hepcidin is an acute-phase protein, the regulation of its transcription should be deeply known when inflammation and anomalies of iron metabolism are contemporary present to allow a better interpretation of the results of the dosages in different cohorts of patients. UC and CD patients could have some little differences in hepcidin expression that should be studied in dedicated clinical trials to possibly obtain some type of peculiar information regarding the anemia of these subtypes of patients.

## 5. Conclusions

In conclusion, it is necessary to intensify research and academic discussion in order to establish what laboratory tests must be included in the clinical assessment of anemic IBD patients, especially in order to differentiate IDA from ACD or diagnose their associated presence. The differential diagnosis may be of some help also in the therapeutic approach because iron treatment works better in IDA, while ACD may resolve itself when the disease is under control. When IDA and ACD are contemporary, the first challenge is to correctly diagnose this condition, and it would be important to design a targeted and useful clinical trial to define the best diagnostic process and the most useful treatment.

## Figures and Tables

**Table 1:** General Study information.

Authors Ref.	Year	Country	Study Design/ Number of Centers	Funding Source
Arnold et al. [22]	2009	United Kingdom	Prospective/monocentric	None declared
Oustamanolakis et al. [18]	2011	Greece	Prospective/monocentric	None declared
Bergamaschi et al. [26]	2013	Italy	Prospective/monocentric	Grant from IRCCS Policlinico S. Matteo, Pavia, Italy
Mecklenburg et al. [27]	2014	Switzerland	Observational/Multicentric	Swiss National Science Foundation (33CSC0_134274)
Martinelli et al. [19]	2016	Italy	Cross-sectional/monocentric	Partially supported by the Italian Ministry of Health, Grant RF-2010-2312048 and by General and Specialist Surgery at Second University of Naples (Grant on Normal and Pathological Hematopoiesis)
Krawiec et al. [23]	2017	Poland	Prospective/monocentric	Grant No. MNsd466 Medical University of Lublin
Moran-Lev et al. [20]	2019	Israel	Prospective/monocentric	No financial support
Shu et al. [21]	2019	China	Prospective/monocentric	Grant from National Key R&D Program of China (2018YFC1705400) and the National Natural Science Foundation of China (81630017, 91740117)
Aksan et al. [28]	2020	Germany	Prospective/monocentric	Supported by Vifor Pharma
Stojkovic Lalosevic et al. [24]	2020	Serbia	Case-Control study, Prospective/monocentric	Grant No. OI175030 from the Ministry of Education, Science and Technological Development, Republic of Serbia.
Ben-David et al. [25]	2022	Israel	Prospective/monocentric	None declared
Loveikyte et al. [29]	2023	The Netherlands	Retrospective/monocentric	The research position of R.L. was supported by the Initiative on Crohn’s and Colitis and Cablon Medical. The research position of A.R.B. was supported by an MD-PhD trajectory grant (grant no. 17-57) from the Junior Scientific Masterclass of the University of Groningen, The Netherlands.

**Table 2:** The patient descriptions and clinical settings.

Authors Ref.	Sample Size (No. of Patients) Mean/Median Age (Year)	Gender F%	Controls Mean/Median Age (Year)	Gender F%	No. of Patients and Clinical Setting	IBD Subtype (No. of Patients)	Comparative Test
Arnold et al. [22]	61 44.03	n.a.	25 36.02	n.a. *	18 anemic 41 non-anemic	10 CD 51 UC	Ferritin IL-6 sTFR
Oustamanolakis et al. [18]	100 49	42%	102 49	19.61	n.a.	51 CD 49 UC	Ferritin CRP
Bergamaschi et al. [26]	54 46	44%	36 43	50	26 anemic 26 non-anemic	22 CD 32 UC	Ferritin CRP sTFR
Mecklenburg et al. [27]	247 36.7	45%	21 n.a.	n.a.	102 anemic 145 non-anemic	130 CD 117 UC	Ferritin CRP
Martinelli et al. [19]	50 12.6	46%	50 11.1	50%	12 anemic 38 non-anemic	21 CD 29 UC	Ferritin CRP sTFR
Krawiec et al. [23]	75 12.9	46.7%	21 11.2	38.1%	38 anemic 37 non-anemic	29 CD 46 UC	Ferritin
Moran-Lev et al. [20]	40 14.8	50%	45 13.5	47%	n.a.	23 CD 17 UC	Ferritin CRP IL-6
Shu et al. [21]	99 40	47.5%	22 39.75	40.9%	59 anemic 40 non-anemic	66 CD 33 UC	Ferritin CRP ESR Serum iron
Aksan et al. [28]	73 36.4	46.6%	22 33.8	50%	20 anemic 53 non-anemic	41 CD 32 UC	Ferritin CRP sTFR
Stojkovic Lalosevic et al. [24]	45 47.4	40%	n.a. n.a.	n.a.	45 anemic 0 non-anemic	22 CD 23 UC	Ferritin CRP ESR
Ben-David et al. [25]	10 15.1	70%	8 10.88	62.5%	10 anemic 0 non-anemic	n.a.	Ferritin CRP sTFR
Loveikyte et al. [29]	122 42.2	48%	50 45	46.4%	52 anemic 70 non-anemic	66 CD 56 UC	Ferritin CRP sTFR ESR

* n.a.: not available.

**Table 3:** The index test characteristics key.

Authors Ref.	Biomarker Patients: Hepcidin ng/mL	*p* Value	Test Type	Main Comparator Patients: Ferritin mg/L	Correlation
Arnold et al. [22]	Mean ± SEM Patients 5.45 ± 0.96 * Controls 15.35 ± 3.14	0.0045	RIA	Patients n.a. Controls n.a.	Pearson’s correlation r = 0.73 *p* < 0.0001
Oustamanolakis et al. [18]	Median (range) Patients 72.1 (16.6–521) * Controls 47.0 (8.6–340.2)	<0.0001	ELISA	Median (range) Patients: 71.0 (10.0–259.5) Controls 37.8 (2.9–447).	Multivariate r = 0.34 *p* = 0.0008
Bergamaschi et al. [26]	Geometric means and 95% CIs (nM) Patients 1.88 (1.12–3.17) 5.24 (3.12–8.84) ng/mL Controls 3.62 (2.86–3.62) 10.09 (7.98–10.09) ng/ml	N.S. ^§^	Mass spectrometry	Median (range) Patients 45 (34–360) Controls 62 (48–79)	Linear regression r = 0.645 *p* = <0.001
Mecklenburg et al. [27]	Median (range) Patients Controls	N.S.	ELISA	Median (range) Patients Controls	Multi-linear regression r = n.a. *p* = 0.005
Martinelli et al. [19]	Mean ± SD (range) Patients 4.5 ± 8.6 (0.55–38.9) nM 12.55 ± 24.0 (1.5–108.5) ng/mL Controls 2.1 ± 2.6 (0.55–11.3) nM 5.86 ± 7.25 (1.5–31.5) ng/mL	0.0623	Mass spectrometry	Mean ± SD (range) Patients 45 ± 36.8 (6–217) Controls 56.8 ± 31 (10–179)	Correlation log-transformed r = 0.442 *p* = 0.0001
Krawiec et al. [23]	Mean ± SD, median (range) Patients 5.9 ± 5.08, 4.76 (0.27–32.93) * Controls 10.00 ± 10.04, 7.15 (0.004–44.78)	<0.03	ELISA	Mean ± SD, median (range) Patients 33.94 ± 39.78, 17.50 (1.00–146.00) Controls 47.52 ± 37.04, 34.00 (8.00–158.00)	Spearman’s correlation r = 0.32 *p* = 0.007
Moran-Lev et al. [20]	Median (interquartile range) Patients 34.2 (21.3–44.7) Controls 13.3 (3.5–23.4)	<0.01	ELISA	Median (interquartile range) Patients 27 (11–55) Controls n.a.	No significant differences in ferritin concentration between the two groups
Shu et al. [21]	Median (range) Patients n.a. Controls n.a.	<0.001 active pathology vs. controls No differences between remission and controls	ELISA	Median (range) Patients n.a. Controls n.a.	Spearman’s correlation UC patients r = 0.8172 *p* = <0.0001 CD patients r = 0.4661 *p* = 0.0002 no cumulative correlation
Aksan et al. [28]	Median (range) Patients 21.95 (11.35–158.05) Controls 26.45 (16.80–56.2)	n.a.^†^	n.a.	Median (range) Patients 51.35 (5.45–791) Controls 82.90 (4.38–300.00)	Spearman’s correlation r = 0.657 *p* < 0.001
Stojkovic Lalosevic et al. [24]	Mean ± SD Patients 6.40 ± 2.42 Controls 9.77 ± 2.71	0.001	ELISA	Mean ± SD Patients 119 ± 124 Controls 394 ± 515	Pearson’s correlation r = n.a. *p* = n.a. generic positive correlation
Ben-David et al. [25]	Mean ± SD Patients 6.8 ± 3.6 Controls (celiac disease) 6.6 ± 5.9	0.93	n.a.	Mean ± SD Patients 18.4 ± 17 Controls (celiac disease) 9.0 ± 6.9	No specified type of correlation r = 0.657 *p* = 0.05
Loveikyte et al. [29]	Median (range) Patients 13.52 (4.85–28.72) Controls 21.19 (9.84–33.29)	0.14	ELISA	Median (range) Patients 45.50 (23.75–92.00) Controls 82.50 (44.00–211.25)	Spearman’s rank correlation coefficients (ρ) Rho = 0.74 *p* < 0.01

* For reasons of space, the mean of the two values of the patients is reported in this Table. ^§^ N.S. Non-significant. ^†^ Not available.

## Data Availability

The data presented in this study are available on request from the corresponding author.

## Supplementary Materials

The following supporting information can be downloaded at: https://www.mdpi.com/article/10.3390/diagnostics14040375/s1, Supplemental Table S1: PRISMA_2020_checklist.

## References

[1] Podolsky D.K. (2002). Inflammatory bowel disease. N. Engl. J. Med..

[2] Mahadea D., Adamczewska E., Ratajczak A.E., Rychter A.M., Zawada A., Eder P., Dobrowolska A., Krela-Kaźmierczak I. (2021). Iron Deficiency Anemia in Inflammatory Bowel Diseases—A Narrative Review. Nutrients.

[3] Aksan A., Beales I.L.P., Baxter G., de Arellano A.R., Gavata S., Valentine W.J., Hunt B. (2021). Evaluation of the Cost-Effectiveness of Iron Formulations for the Treatm of Iron Deficiency Anaemia in Patients with Inflammatory Bowel Disease in the UK. Clin. Outcomes Res..

[4] Ng S.C., Shi H.Y., Hamidi N., Underwood F.E., Tang W., Benchimol E.I., Panaccione R., Ghosh S., Wu J.C.Y., Chan F.K.L. (2017). Worldwide incidence and prevalence of inflammatory bowel disease in the 21st century: A systematic review of population-based studies. Lancet.

[5] Hershko C. (2018). Assessment of iron deficiency. Haematologica.

[6] Torres J., Halfvarson J., Rodríguez-Lago I., Hedin C.R.H., Jess T., Dubinsky M., Croitoru K., Colombel J.F. (2021). Results of the Seventh Scientific Workshop of ECCO: Precision Medicine in IBD-Prediction and Prevention of Inflammatory Bowel Disease. J. Crohns Colitis.

[7] Ransford R.A., Langman M.J. (2002). Sulphasalazine and mesalazine: Serious adverse reactions re-evaluated on the basis of suspected adverse reaction reports to the Committee on Safety of Medicines. Gut.

[8] Eriksson C., Henriksson I., Brus O., Zhulina Y., Nyhlin N., Tysk C., Montgomery S., Halfvarson J. (2018). Incidence, prevalence and clinical outcome of anaemia in inflammatory bowel disease: A population-based cohort study. Aliment. Pharmacol. Ther..

[9] Woźniak M., Barańska M., Małecka-Panas E., Talar-Wojnarowska R. (2019). The prevalence, characteristics, and determinants of anaemia in newly diagnosed patients with inflammatory bowel disease. Prz. Gastroenterol..

[10] Mücke V., Mücke M.M., Raine T., Bettenworth D. (2017). Diagnosis and treatment of anemia in patients with inflammatory bowel disease. Ann. Gastroenterol..

[11] Hentze M.W., Muckenthaler M.U., Galy B., Camaschella C. (2010). Two to tango: Regulation of Mammalian iron metabolism. Cell.

[12] Ganz T. (2019). The Discovery of the Iron-Regulatory Hormone Hepcidin. Clin. Chem..

[13] Girelli D., Busti F., Brissot P., Cabantchik I., Muckenthaler M.U., Porto G. (2022). Hemochromatosis classification: Update and recommendations by the BIOIRON Society. Blood.

[14] Biasiotto G., Carini M., Bresciani R., Ferrari F. (2023). Hereditary hemochromatosis: The complex role of the modifier genes. J. Trace Elem. Med. Biol..

[15] Zanella I., Rossini A., Di Lorenzo D., Biasiotto G. (2015). Hereditary hemochromatosis: The same old song. Blood Cells Mol. Dis..

[16] Nemeth E., Ganz T. (2023). Hepcidin and Iron in Health and Disease. Annu. Rev. Med..

[17] Carini M., Fredi M., Cavazzana I., Bresciani R., Ferrari F., Monti E., Franceschini F., Biasiotto G. (2023). Frequency Evaluation of the Interleukin-6 174G>C Polymorphism and Homeostatic Iron Regulator (HFE) Mutations as Disease Modifiers in Patients Affected by Systemic Lupus Erythematosus and Rheumatoid Arthritis. Int. J. Mol. Sci..

[18] Oustamanolakis P., Koutroubakis I.E., Messaritakis I., Malliaraki N., Sfiridaki A., Kouroumalis E.A. (2011). Serum hepcidin and prohepcidin concentrations in inflammatory bowel disease. Eur. J. Gastroenterol. Hepatol..

[19] Martinelli M., Strisciuglio C., Alessandrella A., Rossi F., Auricchio R., Campostrini N., Girelli D., Nobili B., Staiano A., Perrotta S. (2016). Serum Hepcidin and Iron Absorption in Paediatric Inflammatory Bowel Disease. J. Crohns Colitis.

[20] Moran-Lev H., Galai T., Yerushalmy-Feler A., Weisman Y., Anafy A., Deutsch V., Cipok M., Lubetzky R., Cohen S. (2019). Vitamin D Decreases Hepcidin and Inflammatory Markers in Newly Diagnosed Inflammatory Bowel Disease Paediatric Patients: A Prospective Study. J. Crohns Colitis.

[21] Shu W., Pang Z., Xu C., Lin J., Li G., Wu W., Sun S., Li J., Li X., Liu Z. (2019). Anti-TNF-*α* Monoclonal Antibody Therapy Improves Anemia through Downregulating Hepatocyte Hepcidin Expression in Inflammatory Bowel Disease. Mediat. Inflamm..

[22] Arnold J., Sangwaiya A., Bhatkal B., Geoghegan F., Busbridge M. (2009). Hepcidin and inflammatory bowel disease: Dual role in host defence and iron homoeostasis. Eur. J. Gastroenterol. Hepatol..

[23] Krawiec P., Mroczkowska-Juchkiewicz A., Pac-Kożuchowska E. (2017). Serum Hepcidin in Children with Inflammatory Bowel Disease. Inflamm. Bowel Dis..

[24] Stojkovic Lalosevic M., Toncev L., Stankovic S., Dragasevic S., Stojkovic S., Jovicic I., Stulic M., Culafic D., Milovanovic T., Stojanovic M. (2020). Hepcidin Is a Reliable Marker of Iron Deficiency Anemia in Newly Diagnosed Patients with Inflammatory Bowel Disease. Dis. Markers.

[25] Ben-David Y., Koren A., Colodner R., Levin C. (2022). Characterization of acquired anemia in children by iron metabolism parameters. Sci. Rep..

[26] Bergamaschi G., Di Sabatino A., Albertini R., Costanzo F., Guerci M., Masotti M., Pasini A., Massari A., Campostrini N., Corbella M. (2013). Serum hepcidin in inflammatory bowel diseases: Biological and clinical significance. Inflamm. Bowel Dis..

[27] Mecklenburg I., Reznik D., Fasler-Kan E., Drewe J., Beglinger C., Hruz P., Group S.I.C.S. (2014). Serum hepcidin concentrations correlate with ferritin in patients with inflammatory bowel disease. J. Crohns Colitis.

[28] Aksan A., Wohlrath M., Iqbal T.H., Dignass A., Stein J. (2020). Inflammation, but Not the Underlying Disease or Its Location, Predicts Oral Iron Absorption Capacity in Patients with Inflammatory Bowel Disease. J. Crohns Colitis.

[29] Loveikyte R., Bourgonje A.R., van der Reijden J.J., Bulthuis M.L.C., Hawinkels L.J.A.C., Visschedijk M.C., Festen E.A.M., van Dullemen H.M., Weersma R.K., van Goor H. (2023). Hepcidin and Iron Status in Patients with Inflammatory Bowel Disease Undergoing Induction Therapy with Vedolizumab or Infliximab. Inflamm. Bowel Dis..

[30] Page M.J., McKenzie J.E., Bossuyt P.M., Boutron I., Hoffmann T.C., Mulrow C.D., Shamseer L., Tetzlaff J.M., Akl E.A., Brennan S.E. (2021). The PRISMA 2020 statement: An updated guideline for reporting systematic reviews. BMJ.

[31] Whiting P.F., Rutjes A.W., Westwood M.E., Mallett S., Deeks J.J., Reitsma J.B., Leeflang M.M., Sterne J.A., Bossuyt P.M., Group Q. (2011). QUADAS-2: A revised tool for the quality assessment of diagnostic accuracy studies. Ann. Intern. Med..

[32] Lucendo A.J., Arias Á., Roncero Ó., Hervías D., Verdejo C., Naveas-Polo C., Bouhmidi A., Lorente R., Alcázar L.M., Salueña I. (2017). Anemia at the time of diagnosis of inflammatory bowel disease: Prevalence and associated factors in adolescent and adult patients. Dig. Liver Dis..

[33] Dignass A.U., Gasche C., Bettenworth D., Birgegård G., Danese S., Gisbert J.P., Gomollon F., Iqbal T., Katsanos K., Koutroubakis I. (2015). European consensus on the diagnosis and management of iron deficiency and anaemia in inflammatory bowel diseases. J. Crohns Colitis.

[34] Zanella I., Paiardi G., Di Lorenzo D., Biasiotto G. (2020). Iron Absorption in Celiac Disease and Nutraceutical Effect of 7-Hydroxymatairesinol. Mini-Review. Molecules.

[35] Minhas H.J., Papamichael K., Cheifetz A.S., Gianotti R.J. (2023). A primer on common supplements and dietary measures used by patients with inflammatory bowel disease. Ther. Adv. Chronic Dis..

[36] Camaschella C., Nai A., Silvestri L. (2020). Iron metabolism and iron disorders revisited in the hepcidin era. Haematologica.

[37] Biasiotto G., Ferrari F. (2022). Covidin, a possible new player between hepcidin and ferroportin in hypoxia and inflammation caused by COVID-19. J. Cell Biochem..

[38] Dignass A., Farrag K., Stein J. (2018). Limitations of Serum Ferritin in Diagnosing Iron Deficiency in Inflammatory Conditions. Int. J. Chronic Dis..

[39] Daude S., Remen T., Chateau T., Danese S., Gastin I., Baumann C., Gueant J.L., Peyrin-Biroulet L. (2020). Comparative accuracy of ferritin, transferrin saturation and soluble transferrin receptor for the diagnosis of iron deficiency in inflammatory bowel disease. Aliment. Pharmacol. Ther..

[40] Woźniak M., Borkowska A., Jastrzębska M., Sochal M., Małecka-Wojciesko E., Talar-Wojnarowska R. (2023). Clinical and Laboratory Characteristics of Anaemia in Hospitalized Patients with Inflammatory Bowel Disease. J. Clin. Med..

[41] Kamei D., Nagano M., Takagaki T., Sakamoto T., Tsuchiya K. (2023). Comparison between liquid chromatography/tandem mass spectroscopy and a novel latex agglutination method for measurement of hepcidin-25 concentrations in dialysis patients with renal anemia: A multicenter study. Heliyon.

[42] Oppen K., Brede C., Skadberg Ø., Steinsvik T., Holter J.C., Michelsen A.E., Heggelund L. (2023). Hepcidin analysis in pneumonia: Comparison of immunoassay and LC-MS/MS. Ann. Clin. Biochem..

[43] Delaby C., Vialaret J., Hirtz C., Lefebvre T., Herkert M., Puy H., Lasocki S., Lehmann S., group H.s. (2021). Analytical comparison of ELISA and mass spectrometry for quantification of serum hepcidin in critically ill patients. Bioanalysis.

[44] Weiss G., Ganz T., Goodnough L.T. (2019). Anemia of inflammation. Blood.

[45] Darshan D., Frazer D.M., Wilkins S.J., Anderson G.J. (2010). Severe iron deficiency blunts the response of the iron regulatory gene Hamp and pro-inflammatory cytokines to lipopolysaccharide. Haematologica.

[46] Theurl I., Schroll A., Nairz M., Seifert M., Theurl M., Sonnweber T., Kulaksiz H., Weiss G. (2011). Pathways for the regulation of hepcidin expression in anemia of chronic disease and iron deficiency anemia in vivo. Haematologica.

[47] Weber C.S., Beck-da-Silva L., Goldraich L.A., Biolo A., Clausell N. (2013). Anemia in heart failure: Association of hepcidin levels to iron deficiency in stable outpatients. Acta Haematol..

